# Self-rated health and chronic inflammation are related and independently associated with hospitalization and long-term mortality in the general population

**DOI:** 10.1038/s41598-022-24422-z

**Published:** 2022-11-17

**Authors:** Juliette Tavenier, Line Jee Hartmann Rasmussen, Janne Tolstrup, Janne Petersen, Jakob Sobocki, Charlotta Pisinger, Jesper Eugen-Olsen, Hejdi Gamst-Jensen

**Affiliations:** 1 Department of Clinical Research, Copenhagen University Hospital Hvidovre, Hvidovre, Denmark; 2 Department of Psychology and Neuroscience, Duke University, Durham, NC USA; 3 National Institute of Public Health, University of Southern Denmark, Odense, Denmark; 4 Copenhagen Phase 4 Unit, Department of Clinical Pharmacology and Center for Clinical Research and Prevention, Copenhagen University Hospital Bispebjerg and Frederiksberg, Frederiksberg, Denmark; 5 Section of Biostatistics, Department of Public Health, University of Copenhagen, Copenhagen, Denmark; 6 Emergency Department, Copenhagen University Hospital Hvidovre, Hvidovre, Denmark; 7 Center for Clinical Research and Prevention, Copenhagen University Hospital Bispebjerg and Frederiksberg, Frederiksberg, Denmark; 8 Department of Public Health, Faculty of Health and Medical Sciences, University of Copenhagen, Copenhagen, Denmark

**Keywords:** Biomarkers, Health services, Biomarkers, Epidemiology

## Abstract

The subjective indicator of health self-rated health (SRH) and the chronic inflammation biomarker soluble urokinase plasminogen activator receptor (suPAR) are both robust predictors of healthcare use and mortality. However, the possible relationship between SRH and suPAR in the assessment of hospitalization and mortality risk is unknown. We used data from the Danish population-based Inter99 cohort to examine the association between SRH and suPAR and test their individual and combined associations with 2-year risk of acute hospitalization and 5- and 15-year mortality. SRH and serum suPAR levels were measured in 5490 participants (median age 45.1 years, 48.7% men). Poorer SRH was associated with elevated suPAR. In unadjusted analyses, SRH and suPAR were individually associated with higher risks of acute hospitalization and mortality, and both measures remained independently associated with higher risks of hospitalization and 15-year mortality after mutual adjustments. The association of suPAR with mortality was stronger in poorer SRH categories, and when combined, SRH and suPAR could identify different groups of individuals with increased risk of acute hospitalization and mortality. Both SRH and suPAR were independently associated with risk of acute hospitalization and mortality, and different combinations of the two measures could identify different groups of individuals at increased risk.

## Introduction

There is solid evidence that a person’s self-evaluation of their overall health status is a strong predictor of morbidity, use of health services, and mortality independently of objective measures of health and other risk factors such as socioeconomic status, age, chronic illness, gender, and physical examination^1–6^. Self-rated health (SRH) is a simple subjective measure capturing a condensed summary of experienced somatic information, and is thought to have a biological foundation, however, these processes are not yet well identified^7^.

Studies have suggested that “sickness signals” such as changes in biomarker levels influence how the body or brain interpret general health, and that a deviation from normal values might negatively impact a person’s SRH^8–10^. In a recent study, researchers found a significant association between SRH and 57 out of 150 investigated blood and urine biomarkers encompassing a wide range of biological domains, and 26 of the biomarkers (including the inflammation marker C-reactive protein [CRP]) remained significantly associated with SRH after adjusting for chronic diseases and physical functioning^10^. Several other studies have reported associations between SRH and biomarkers of systemic inflammation, including cytokines^8,11–13^, CRP^10–12^, white blood cell counts^9^. A small number of studies further examined whether biomarker levels explained the strong association between SRH and mortality, and all reported that after adjusting for biomarkers, the association was only slightly weakened and SRH remained independently associated with mortality^9,10,14–16^. Only two of these studies have compared the performance of SRH and biomarkers in the prediction of mortality and found that while SRH was a better predictor of mortality than biomarkers^14,15^, the combination of SRH with biomarkers improved the predictive ability of the models^14^.

The soluble form of the urokinase plasminogen activator receptor (suPAR) is a novel biomarker of systemic chronic inflammation^17^, and a strong, unspecific, prognostic biomarker for morbidity and mortality^18,19^. suPAR levels are elevated with many chronic diseases^18,20,21^, and shown to positively correlate with other inflammatory biomarkers and remain a strong predictor of adverse health outcomes even after adjustments for inflammatory biomarkers such as IL-6 and CRP^18,22–26^. suPAR levels are elevated by unhealthy lifestyle such as smoking and an unhealthy diet^19^. However, change of lifestyle habits towards a healthier lifestyle results in lowering of suPAR, and the resultant suPAR is predictive of mortality^19,27^. Furthermore, a recent study demonstrated that suPAR is a causal factor in the development of cardiovascular disease^28^. This indicates that suPAR is an early warning biomarker and that interventions modifying suPAR can lower the risk of adverse outcomes.

Both SRH and suPAR are unspecific measures of general health and strong prognostic markers of outcome. Poor SRH is correlated with higher suPAR^24,29^, but the relationship between the two biomarkers has not been thoroughly investigated, and it is unclear whether they reflect common or independent processes related to increased risk of illness and mortality. The present study aimed to (i) examine the association between SRH and suPAR, (ii) test whether SRH and suPAR are independently associated with the risk of acute hospitalization and mortality, as well as (iii) test the interaction between SRH and suPAR in their association with the risk of acute hospitalization and mortality.

## Results

### Participant characteristics

**Figure 1:**
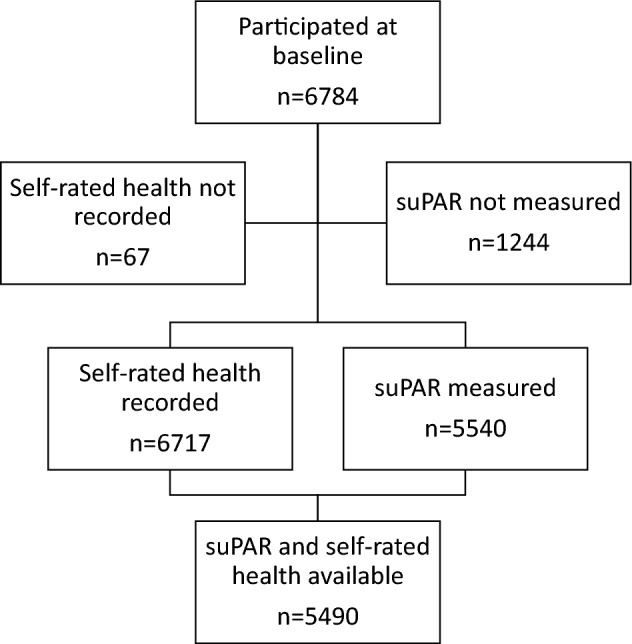
Flowchart of study participants.

Of the 6784 individuals who participated in the baseline examination, 6717 had answered the SRH question, and 5540 had a serum suPAR measurement available. A total of 5490 individuals had data available for both SRH and suPAR levels and were included in this study (Fig. 1). Baseline characteristics of the participants according to the three categories of SRH or suPAR are presented in Table 1. The characteristics of the participants according to the four combinations of good/poor SRH and high/low suPAR are presented in Supplementary Table 1.

**Table 1:** Participant characteristics at baseline and outcomes according to self-rated health or suPAR categories.

	All	Self-rated health				suPAR			
	n = 5490	Excellent/Very good (n = 1830)	Good (n = 3087)	Fair/Bad (n = 573)	*P*	Low (0.649–2.950 ng/mL, n = 1830)	Intermediate (> 2.950– < 5.460 ng/mL, n = 3087)	High (5.460–22 ng/mL, n = 573)	*P*
**Demography**									
Age (years)	45.1 (40.0–50.3)	45.0 (39.9–50.1)	45.1 (40.0–50.4)	45.9 (40.1–54.8)	< .0001	45.0 (39.9–50.1)	45.1 (40.0–54.7)	45.1 (40.1–50.5)	< .0001
Male	2672 (48.7)	926 (50.6)	1498 (48.5)	248 (43.3)	0.009	1100 (60.1)	1340 (43.4)	232 (40.5)	< .0001
Type 2 diabetes (self-reported)	110 (2.0)	19 (1.1)	48 (1.6)	43 (7.6)	< .0001	25 (1.4)	63 (2.1)	22 (3.9)	0.001
Hypertension (self-reported)	1015 (20.2)	245 (14.8)	587 (20.7)	183 (34.6)	< .0001	285 (17.2)	613 (21.6)	117 (22.5)	0.002
**Socio-economic status**					< .0001				< .0001
Low	1127 (22.3)	292 (17.0)	637 (22.6)	198 (37.9)		310 (18.2)	661 (23.4)	156 (29.5)	
Medium	3339 (66.1)	1205 (70.2)	1864 (66.2)	270 (51.7)		1143 (67.1)	1868 (66.2)	328 (62.0)	
High	589 (11.7)	220 (12.8)	315 (11.2)	54 (10.3)		250 (14.7)	294 (10.4)	45 (8.5)	
**Lifestyle**									
Physical activity					< .0001				< .0001
Low	1177 (21.9)	260 (14.4)	704 (23.2)	213 (38.9)		345 (19.2)	673 (10.1)	159 (28.8)	
Light	3300 (61.3)	1076 (59.7)	1933 (63.7)	291 (53.1)		1060 (58.9)	1907 (62.9)	333 (60.3)	
Moderate	847 (15.7)	422 (23.4)	384 (12.7)	41 (7.5)		363 (20.2)	426 (14.1)	58 (10.5)	
High	58 (1.1)	43 (2.4)	12 (0.4)	3 (0.6)		32 (1.8)	24 (0.8)	2 (0.4)	
Smoking					< .0001				< .0001
Never	1949 (35.6)	733 (40.2)	1066 (34.7)	150 (26.5)		850 (46.6)	1033 (33.6)	66 (11.6)	
Former	1374 (25.1)	489 (26.8)	772 (25.1)	113 (19.9)		578 (31.7)	739 (24.0)	57 (10.0)	
Occasional	204 (3.7)	77 (4.2)	107 (3.5)	20 (3.5)		76 (4.2)	121 (3.9)	7 (1.2)	
Daily	1941 (35.5)	526 (28.8)	1131 (36.8)	284 (50.1)		319 (17.5)	1181 (38.4)	441 (77.2)	
Alcohol					< .0001				< .0001
Abstinent	530 (10.1)	127 (7.2)	303 (10.2)	100 (19.2)		148 (8.4)	297 (23.4)	85 (15.8)	
Within recommendations	3895 (74.0)	1409 (79.3)	2156 (72.8)	330 (63.3)		1350 (76.3)	2181 (73.9)	364 (67.4)	
Overuse	836 (15.9)	241 (13.6)	504 (17.0)	91 (17.5)		272 (15.4)	473 (16.0)	91 (16.9)	
BMI (kg/m^2^)	25.6 (23.1–28.6)	24.9 (22.8–27.4)	25.9 (23.3–28.9)	27.0 (23.5–30.2)	< .0001	25.4 (23.0–27.8)	25.9 (23.3–29.1)	25.1 (22.6–29.0)	< .0001

Data are presented as median (interquartile range) or n (%).

### Relationship between SRH and suPAR levels

**Figure 2:**
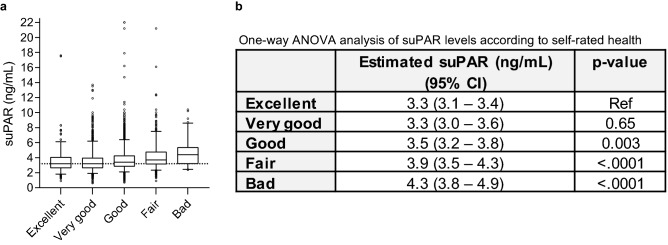
Association between self-rated health and suPAR. (**a**) Box plots of soluble urokinase plasminogen activator receptor (suPAR) levels according to self-rated health. Boxes represent 25th–75th percentiles, whiskers represent 5th–95th percentiles, and the line represents the median suPAR values. The dotted line represents the median suPAR value for the *Excellent* category. (**b**) One-way ANOVA analysis of suPAR levels according to self-rated health. *Excellent* self-rated health is used as reference.

The participants had a median suPAR level of 3.4 ng/mL (IQR: 2.7–4.3), and suPAR levels increased with worse SRH (Fig. 2). When reducing SRH into 3 categories, suPAR levels for participants reporting *excellent/very good* SRH were 3.2 ng/mL (IQR: 2.6–4.0), 3.4 ng/mL (IQR: 2.8–4.3) for those reporting *good* SRH, and 3.8 ng/mL (IQR: 3.1–4.9) for those reporting *fair/bad* SRH (Supplementary Table 2). The number of participants within each of the SRH categories according to their suPAR category is also shown in Supplementary Table 2.

### Individual and mutually adjusted associations of SRH and suPAR with the risk of acute hospitalization

**Figure 3:**
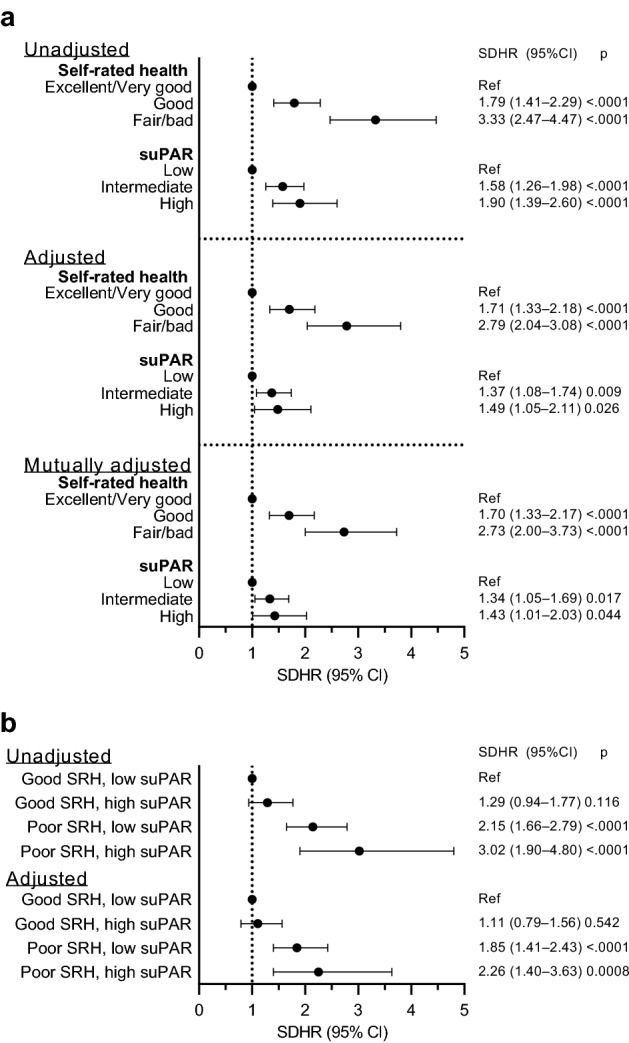
Associations of self-rated health and suPAR levels with acute hospitalization. Sub-distribution hazard ratios (SDHRs; symbols) and 95% confidence intervals (CIs; error bars) for acute hospitalization within 2 years after baseline by self-rated health (SRH) or soluble urokinase plasminogen activator receptor (suPAR) categories. (**a**) 2-year risk of acute hospitalization by self-rated health or suPAR categories, and (**b**) stratified by poor self-rated health and/or high suPAR. The adjusted models include age, sex, smoking, BMI, and comorbidities. The mutually adjusted model includes age, sex, smoking, BMI, comorbidities, and both SRH and suPAR.

Within 2 years after baseline examination, 437 (8.0%) participants were acutely hospitalized, 8 (0.2%) had died, and 10 (0.2%) were lost to follow-up. When suPAR levels were analyzed as a continuous variable, a doubling in suPAR levels was associated with a significantly higher risk of acute hospitalization both in unadjusted (Sub-distribution HR: 1.50, 95% CI: 1.27 to 1.78, *P* < 0.0001) and adjusted (SDHR: 1.30, 95% CI: 1.06 to 1.59, *P* = 0.011) analyses. The interaction between suPAR and sex was not statistically significant (*P* = 0.46). In individual analyses of SRH and suPAR as 3-level categories, participants reporting *good* or *fair/bad* SRH, as well as those with *intermediate* or *high* suPAR, were at significantly higher risk of hospitalization compared to participants with *excellent/very good* SRH or *low* suPAR, respectively (Fig. 3A). Mutual adjustment for SRH and suPAR did not affect the strength of the associations (Fig. 3A). To further investigate whether SRH and suPAR were independently associated with the risk of acute hospitalization, we added an interaction term between suPAR and SRH categories to the mutually adjusted model. The interaction term was not statistically significant, suggesting that the associations of suPAR or SRH categories with the risk of acute hospitalization are not significantly dependent on each other (*P* = 0.62).

### Combined associations of SRH and suPAR with the risk of acute hospitalization

We also studied the combined associations of SRH and suPAR with the risk of acute hospitalization by stratifying the participants into four or nine groups based on good or poor SRH and high or low suPAR. For participants reporting good SRH, having high suPAR levels did not increase the risk of hospitalization compared to having low suPAR (Fig. 3B). However, poor SRH increased the risk of acute hospitalization for participants with low suPAR, but also for those with high suPAR. The combination of poor SRH and high suPAR presented the highest sub-distribution hazard ratios for hospitalization risk, although CIs overlapped with other combinations (Fig. 3B). Analyses according to the nine combinations of SRH and suPAR groups are presented in Supplementary Table 3.

### Individual and mutually adjusted associations of SRH and suPAR with mortality

**Figure 4:**
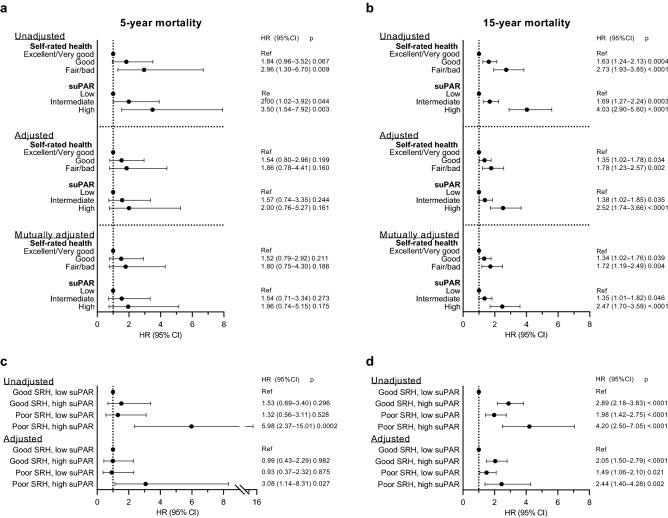
Association of self-rated health and suPAR with mortality. Hazard ratios (HRs; symbols) and 95% confidence intervals (CIs; error bars) for 5-year and 15-year mortality according to self-rated health (SRH) or soluble urokinase plasminogen activator receptor (suPAR) categories. (**a**) 5-year and (**b**) 15-year mortality by self-rated health or suPAR categories. (**c**) 5-year and (**d**) 15-year mortality stratified by poor self-rated health and/or high suPAR. The adjusted model includes age, sex, smoking, BMI, and comorbidities. The mutually adjusted model includes age, sex, smoking, BMI, comorbidities, and both SRH and suPAR.

**Figure 5:**
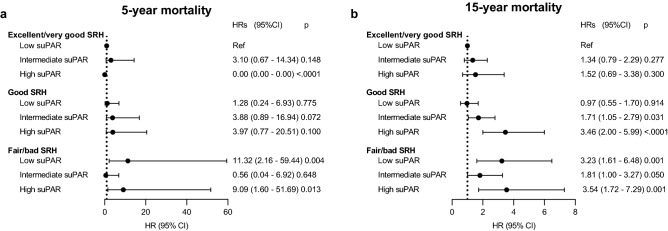
Interaction analyses for the association of suPAR with mortality within each self-rated health category. Hazard ratios (HRs; symbols) and 95% confidence intervals (CIs; error bars) for the association of soluble urokinase plasminogen activator receptor (suPAR) with the risk of (**a**) 5- and (**b**) 15-year mortality within each self-rated health (SRH) category (interaction analysis in the mutually adjusted models). HRs for each level of suPAR and SRH in comparison to the reference group (low suPAR and excellent/very good SRH) group are plotted. Analyses are adjusted for age, sex, smoking, BMI, and comorbidities.

Five years after the baseline examination, 60 (1.1%) participants had died and 19 (0.4%) were lost to follow-up. A doubling in suPAR levels was associated with a significantly higher risk of 5-year mortality in unadjusted (HR: 2.03, 95% CI: 1.24 to 3.33, *P* = 0.005) analyses, but not in analyses adjusted for age, sex, smoking status, BMI, and comorbidities (HR: 1.47, 95% CI: 0.78 to 2.76, *P* = 0.23). The interaction between suPAR levels and sex was not statistically significant (*P* = 0.79). In unadjusted analysis of SRH categories, participants reporting *fair/bad* SRH, but not those reporting *good* SRH, had a significantly higher risk of 5-year mortality compared to participants with *excellent/very good* SRH (Fig. 4A). For suPAR categories, participants with *intermediate* and *high* suPAR had a significantly higher risk of 5- year mortality compared to those with *low* suPAR (Fig. 4A). After controlling for age, sex, smoking status, BMI, and comorbidities as well as in the mutually adjusted model, the associations no longer reached statistical significance (Fig. 4A). In the mutually adjusted model, the interaction terms for suPAR and SRH categories was statistically significant (*P* < 0.0001; Fig. 5A).

Fifteen years after the baseline examination, 326 (5.9%) participants had died and 47 (0.9%) were lost to follow-up. A doubling in suPAR levels was significantly associated with a higher risk of 15-year mortality both in unadjusted (HR: 2.32, 95% CI: 1.89 to 2.85, *P* < 0.0001) and adjusted (HR: 1.81, 95% CI: 1.40 to 2.33, *P* < 0.0001) analyses. When the interaction term between suPAR levels and sex was included (*P* = 0.03), higher suPAR levels were significantly associated with a higher risk of 15-year mortality for men (HR: 2.17, 95% CI: 1.60 to 2.95, *P* < 0.0001), but not for women (HR: 1.29, 95% CI: 0.86 to 1.92, *P* = 0.22). In univariate analyses of SRH or suPAR categories, participants with *good* and *fair/bad* SRH as well as those with *intermediate* and *high* suPAR had a significantly higher risk of 15-year mortality compared to participants with *excellent/very good* SRH or *low* suPAR, respectively (Fig. 4B). The associations remained statistically significant after adjustments for age, sex, smoking, BMI, and comorbidities and mutual adjustments for suPAR and SRH only slightly weakened the associations (Fig. 4B). The interaction terms for suPAR and SRH categories was statistically significant (*P* = 0.02). Figure 5B shows the HRs for each suPAR category within each SRH category, with *low* suPAR in the *excellent/very good* SRH category as the reference. Increasing suPAR levels had a larger effect on the risk of 15-year mortality for participants reporting *good* and *fair/bad* SRH than for those reporting *excellent/very good* SRH (Fig. 5B).

### Combined associations of SRH and suPAR with mortality

When stratifying the participants according to the four combinations of SRH and suPAR groups, only those with both poor SRH and high suPAR had a significantly higher risk of 5-year mortality compared to those with good SRH and low suPAR (Fig. 4C). For 15-year mortality, compared to participants with both good SRH and low suPAR, participants with either poor SRH or high suPAR had a higher risk of 15-year mortality, and the combination of both poor SRH and high suPAR presented the highest hazard ratios, although CIs overlapped with other combinations (Fig. 4D). Analyses according to the nine combinations of SRH and suPAR groups are presented in Supplementary Table 3.

## Discussion

In this large population-based study, we tested the individual and combined associations of a subjective indicator (SRH) and an objective biomarker (suPAR) of health with the risk of acute hospitalization, short- and long-term mortality. The main finding of our study is that both SRH and suPAR were strongly and independently associated with 2-year risk of acute hospitalization and long-term mortality after adjustments for age, sex, smoking, BMI, and comorbidities, and that the strength of these associations was unchanged by mutual adjustments for suPAR and SRH, respectively. Furthermore, we found that overall, different combinations of poorer SRH and/or elevated suPAR were associated with higher risks of acute hospitalization and mortality compared to the combination of the best category of SRH and the lowest suPAR levels.

Similar to findings with other inflammation biomarkers^8–13,30–32^, and previous reports on suPAR^24,29^, we found that suPAR levels increased with poorer SRH. Kananen et al. proposed three pathways by which biomarkers could influence SRH^10^. First, individuals may be aware of their levels of certain biomarkers related to specific diagnoses such as cholesterol or glucose levels and may take these biomarkers-or the associated diagnoses-into account in their assessment of health^10^. Although participants would not have been aware of their suPAR levels at the time of SRH assessment, it is possible that they would take into account other diagnoses or biomarker levels that are known to be associated with elevated suPAR, such as diabetes, cardiovascular disease, elevated cholesterol^18,33–35^. Second, individuals may consider lifestyle and behavioral factors in their health assessment^10^. We have not tested whether lifestyle factors influenced the relationship between suPAR and SRH. But this is a possibility as suPAR levels are known to be dependent on lifestyle^36^ and can be affected by lifestyle changes^19^. Third, changes in biomarker levels can influence the perception of health, and in particular, changes in biomarkers of inflammation including cytokines are thought to be involved in symptoms of sickness such as fatigue, loss of appetite, and weakness^10,37–39^. This mechanism has been suggested to explain part of the relationship between SRH and mortality^10^.

The individual relationships of poor SRH and elevated suPAR levels with risk of acute hospitalization have been described^5,6,40–42^, but the two measures have not previously been compared or combined. In the present study, poor SRH and high suPAR were associated with a higher risk of acute hospitalization independently of each other, though the effect of poor SRH was greater than that of high suPAR. Furthermore, poor SRH was significantly associated with a higher risk of acute hospitalization when combined with either high or low suPAR, whereas high suPAR did not increase risk for individuals with good SRH. Although both SRH and suPAR reflect disease processes, the stronger association of SRH with hospitalization risk may not be surprising as individuals faring badly, for example perceiving a greater illness burden and impaired function, may be more likely to actively seek care and be acutely hospitalized, while elevated chronic inflammation might more strongly reflect slowly developing pathological processes leading to increased biological aging and long-term adverse outcomes. In older adults, a composite score of inflammation (based on albumin, cholesterol, CRP, and IL-6) was a better predictor of mortality than of hospitalization over a 4-year follow-up^43^. A large survey investigating the association of SRH with healthcare use concluded that the feeling of poor health was more strongly associated with risk of hospitalization than diagnosed chronic conditions or age^44^. Thus, the choice to actively seek medical care may be more guided by interoceptive signals or worries, and other behaviors, that are not directly informed by objective health indicators, including chronic inflammation. Nevertheless, poor SRH was also strongly associated with 5- and 15-year mortality, indicating that the feeling of poor health is also grounded in real risk of mortality.

We observed strong associations of poor SRH and high suPAR with mortality, which have also been repeatedly reported in previous studies^2,4,18^. A small number of studies investigated whether the SRH-mortality association was explained by biomarkers such as CRP or panels that included CRP; in all these studies, adjustment for biomarkers only slightly weakened the associations, and SRH remained statistically associated with mortality^10,14–16^. In the present study, the HRs for SRH and mortality risk were largely unaffected when controlling for suPAR. Similarly, the relationship of suPAR with mortality was unaffected after adjusting for SRH. Thus, our—and previous—findings suggest that the association of SRH with mortality cannot be explained by biomarkers, such as suPAR, alone; and the association between biomarkers such as suPAR and mortality cannot be explained by SRH alone^7,15^. Furthermore, in contrast to the reports that SRH is more strongly associated with mortality than biomarker panels^14,15^, in our study, the association of high suPAR with mortality was larger than that of fair/bad SRH. Recent evidence suggests that in contrast to traditional inflammation biomarkers such as CRP and cytokines that reflect acute inflammation, suPAR levels increase and decrease at a slower speed, are less affected by acute changes, and are thought to capture other aspects of chronic inflammation^17^. It is also still unknown whether suPAR is involved in sickness behavior to the same extent as cytokines and acute-phase proteins. Finally, we found that a combination of both suPAR and SRH provided greater information about the risk of mortality than either measure alone; with suPAR adding information about the risk of mortality for participants with excellent to good SRH, and likewise, poorer SRH could identify participants with low suPAR who were at a higher risk of mortality. Furthermore, higher suPAR and poor SRH appeared to impact the risk of 15-year mortality in a synergistic fashion-for participants with good to bad SRH, the risk of mortality further increased with increasing suPAR. Therefore, even though SRH and suPAR levels were related, in this study, the two measures appear to provide unique information regarding health status and may reflect independent pathways to mortality.

Despite being a validated research tool and simple to administer, SRH is not widely used in clinical settings. Incorporating assessment of patient-reported outcomes such as SRH in routine care may help identify at-risk individuals that may otherwise be overlooked by routine biochemical evaluation alone, and those whose complaints during examination are incorrectly interpreted by healthcare professionals. In our study, poor SRH alone, or high suPAR alone, identified different groups of participants, with different risks of hospitalization and mortality, and the combination of both poor SRH and suPAR was consistently associated with an increased risk for all outcomes. Furthermore, in interaction analyses, we found that suPAR was more strongly associated with mortality for participants reporting poor SRH. Thus, the combination of the two biomarkers provided synergistic information on the health status, compared to each measure alone. This may have clinical implications in future intervention studies in the general population, allowing for intervention in this particularly high-risk group of individuals. It has previously been shown that a lifestyle change in the Inter99 cohort, i.e., smoking cessation resulted in lowering of suPAR levels (from baseline to 5-year samples) and that the resultant 5-year suPAR value was associated with future mortality^19^. A recent clinical trial used elevated suPAR levels as a guide for identifying patients with increased risk of progression of COVID-19 and showed a decreased length of hospitalization and mortality for these patients after treatment with an interleukin-1 inhibitor^45^. This indicates that early identification and intervention of high-risk individuals can lead to lowering of risk of unplanned hospitalization and mortality. By combining suPAR and SRH data in the present study, we have significantly enhanced risk identification, possibly leading to a smaller, but more targeted, group of individuals that may be offered intervention (e.g., only those with poor SRH and high suPAR) in future general population intervention studies.

This study has limitations. First, despite a large sample size, this general population cohort was relatively young and healthy, and only 60 (1.1%) participants died during the first 5 years after data collection. Second, other biomarkers of inflammation, such as CRP and cytokines, could have been included in the study for comparison with suPAR as it has been suggested that suPAR and CRP or cytokines may reflect different aspects of inflammation^17^. Third, due to the cross-sectional design of this study, we could not determine whether there was a causal relationship between high suPAR levels and poorer SRH, nor the direction of this relationship, or whether the association between the two could be related to sickness behavior.

Our results show that individuals with poor SRH, high suPAR, or a combination of both poor SRH and high suPAR had increased risk for acute hospitalization and long-term mortality. These findings contribute to the body of knowledge promoting the combined use of subjective and objective indicators of health to assess the risk of healthcare use and mortality.

## Methods

### Study and participants

This study is based on data collected from participants from the Danish Inter99 cohort, a non-pharmacological intervention study for the prevention of ischemic heart disease consisting of 13,016 participants aged 30–60 years who were randomized to either a high or a low intervention of lifestyle counselling. Of these, 6784 agreed to participate and underwent a baseline examination between March 1999 and January 2001^46^. The examination included a clinical examination, extensive questionnaires assessing lifestyle, socioeconomic status, mental health, and SRH, as well as blood sampling. Participants were linked to the Danish health and social registers containing information on hospitalizations, ICD-10 codes, and death^46,47^. The study was conducted in accordance with the Declaration of Helsinki and was approved by the Scientific Ethics Committee of the Capital Region of Denmark (KA 98 155) and the Danish Data Protection Agency, and was registered as a clinical trial (ClinicalTrials.gov; NCT00289237). All participants provided written informed consent before taking part in the study. No effect of the lifestyle intervention was observed in the original study^47^, and we included all participants who had data available for both SRH and suPAR levels in the present study (n = 5490, Fig. 1).

### Self-rated health

SRH was assessed at baseline via a self-administered questionnaire. The question was formulated as: “*How do you think your health is, all in all?*”, with the following possible answers: “*excellent*”, “*very good*”, “*good*”, “*fair*”, or “*bad*”.

### suPAR

Serum levels of suPAR (ng/mL) at baseline were measured using the suPARnostic ELISA assay (Virogates, Birkeroed, Denmark) as previously reported^36^. Samples were measured in singlets, but according to the manufacturer of the suPARnostic ELISA, the intra‐assay variation is 2.8%, and the inter-assay variation as 9.2%. Samples with values below the detectable range of the assay (0.6 ng/mL) were excluded (n = 13), and samples with values above the detectable range were assigned a value corresponding to the upper limit of quantification (22 ng/mL, n = 2).

### Socioeconomic and lifestyle variables, and comorbidities

The participants’ socioeconomic status, smoking, alcohol consumption, and physical activity habits were assessed at baseline via self-administered questionnaires. The socioeconomic status of the participants was based on the duration of their education after primary school and categorized as low (< 2 years), medium (2–4 years), or high (> 4 years)^47^. Smoking was categorized as: never smoker, former smoker, occasional, or daily smoker^36^; alcohol consumption as abstinent (0 units per week), within recommendations (1–21 weekly units for men, or 1–14 for women), or overuse (> 21 weekly units for men, or > 14 units for women)^36^, according to recommendations on alcohol consumption at the time of assessment; and physical activity as low (mainly sedentary), light (walks, biking, or light activity for up to 4 h per week), moderate (moderate activity at least 3 times per week), or high (competitive sports or long-distance running several times per week)^19^. Body mass index (BMI, kg/m^2^) was calculated from weight and height measurements without shoes. Data on comorbidities included self-reported diagnoses of hypertension, diabetes and cardiovascular disease other than myocardial infarction. A variable for comorbidities including diagnoses of diabetes and cardiovascular disease was generated.

### Outcome variables

Participants were followed from the time of baseline examination (March 1999 to January 2001) until 31 December 2016, for the outcomes of acute hospitalization within 2 years as well as 5- and 15-year mortality. Acute hospitalizations within 2 years following baseline examination were identified from the Danish National Patient Register (NPR) as “admission type” coded as acute admissions, and “patient type” coded as 24 h patient, and the time elapsed between the acute hospitalization dates and the date of baseline examination was calculated. If participants had several acute hospitalizations, the acute hospitalization date with the shortest time elapsed after the baseline examination date was selected. Hospitalizations for *Injury, poisoning, and certain other consequences of external causes* (ICD10 codes: S00-T98) as well as *Pregnancy, childbirth, and the puerperium* (ICD10 codes: O00-O99) were excluded. Survival status was obtained from the vital status and date of change in vital status variables from the Danish Civil Registration System (CPR). Survival time for participant with vital status coded as “dead” was calculated as the time elapsed between the baseline examination date and the date of death recorded in the register.

### Statistical analysis

Descriptive data of participants are presented as the median and interquartile range (IQR) for continuous variables or the count and percentage for categorical variables.

We explored the relationship between SRH and suPAR using a one-way ANOVA for comparison of suPAR levels between the SRH category “excellent” and the other four categories. suPAR levels were log-transformed using log2, and back-transformed estimates are reported.

We used Cox regression analyses to assess the independent and combined associations of SRH and suPAR with the risk of acute hospitalization within 2-year after baseline, and of 5- and 15-year mortality. Loss to follow-up was considered a censored event, and for the analysis of acute hospitalization risk, death was considered a competing event. For the analyses of acute hospitalization risk, we report sub-distribution hazard ratio (SDHR) and 95% confidence intervals (CI) obtained using the Fine and Gray competing risk regression models. For mortality risk analyses, we report hazard ratios (HRs) with 95% confidence intervals (CIs).

To assess the independent associations of SRH and suPAR with the outcomes, suPAR was log2-transformed for analysis as a continuous variable and tested in unadjusted models and models adjusted for age, sex, smoking, BMI, and comorbidities; the interaction between suPAR levels and sex was also tested in the adjusted models. The SRH measure was reduced to 3 categories: *excellent/very good* (n = 1830, 33.3%), *good* (n = 3087, 56.2%), and *fair/bad* (n = 573, 10.4%). Clinical cut-offs for suPAR have not yet been established. Therefore, and to ease comparison between SRH and suPAR, we created a categorical variable for suPAR where participants were stratified into three categories of the same size as the three SRH categories: *low suPAR* (range: 0.649–2.950 ng/mL, n = 1830, 33.3%), *intermediate suPAR* (> 2.950–< 5.460 ng/mL, n = 3087, 56.2%), and *high suPAR* (5.460–22 ng/mL, n = 573, 10.4%). The associations of 3-level SRH and suPAR were tested individually in unadjusted models and models adjusted for age, sex, smoking, BMI and comorbidities, as well as in a mutually adjusted model that included both the 3-level SRH and suPAR, age, sex, smoking, BMI and comorbidities. In addition, we tested the associations of suPAR categories with the outcomes within each of the SRH categories by adding an interaction term for SRH and suPAR in the mutually adjusted models.

Finally, to assess the individual and combined associations of SRH and suPAR with the outcomes, we stratified the participants into four groups: (i) good SRH and low suPAR, n = 4434 (80.8%), (ii) good SRH and high suPAR, n = 483 (8.8%); (iii) poor SRH and low suPAR, n = 483 (8.8%); and (iv) poor SRH and high suPAR, n = 90 (1.6%). The good SRH group consisted of participants who answered “*excellent*”, “*very good*”, or “*good*” (n = 4917, 89.6%), and the poor SRH group consisted of participants who answered “*fair*” or “*bad*” (n = 573, 10.4%) to the questionnaire. The low suPAR group consisted of participants from the previously defined *low* and *intermediate* suPAR categories (n = 4917, 86.6%), and the high suPAR group consisted of participants in the previously defined *high* suPAR category (n = 573, 10.4%). Furthermore, we stratified the participants into nine groups (based on the 3 levels of SRH and the 3 levels of suPAR). We tested the associations of the four and the nine combined SRH and suPAR groups with the outcomes in unadjusted and adjusted (age, sex, smoking, BMI, and comorbidities) models.

For statistical analysis, we used SAS Enterprise Guide version 7.15 (SAS Institute, Cary, NC, USA). Graphs were made in GraphPad Prism version 9 (GraphPad Software Inc., San Diego, CA, USA). Two-sided p-values are reported, and statistical significance was defined as a p-value < 0.05.

## Supplementary Information

Supplementary Information.

## Data Availability

The datasets are not publicly available due to regulations set out by the Danish Data Protection Agency but are available from J.P. on reasonable request.
