# Supplementary material for: Self-rated health and chronic inflammation are related and independently associated with hospitalization and long-term mortality in the general population
Source: Sci Rep. 2022 Nov 17;12:19761. doi: 10.1038/s41598-022-24422-z (PMC9670062; doi:10.1038/s41598-022-24422-z)
Supplement: Supplementary file 1 — Supplementary Information. [file 41598_2022_24422_MOESM1_ESM.pdf]

Self-rated health and chronic inflammation are related and independently associated with hospitalization and long-term mortality in the general population

Juliette Tavenier[1*], Line Jee Hartmann Rasmussen[1,2], Janne Tolstrup[3], Janne Petersen[4,5], Jakob Sobocki[6], Charlotta Pisinger[7,8], Jesper Eugen-Olsen[1], Hejdi Gamst-Jensen[1,6]

Supplementary information

Supplementary Table 1. Participant characteristics at baseline and outcomes according to self-rated health and suPAR level combinations

	**Excellent–good SRH, low suPAR (n=4434, 80.8%)**	**Excellent–good SRH, high suPAR (n=483, 8.8%)**	**Fair/bad SRH, low suPAR (n=483, 8.8%)**	**Fair/bad SRH, high suPAR (n=90, 1.6%)**
**Demography**				
Age (years)	45.1 (39.9 – 50.2)	45.1 (40.1 – 50.2)	45.6 (40.1 – 54.8)	49.7 (40.1 – 54.9)
Male	2226 (50.2)	198 (41.0)	214 (44.3)	34 (37.8)
Type 2 diabetes (self-reported)	57 (1.3)	10 (2.1)	31 (6.5)	12 (13.6)
Hypertension (self-reported)	748 (18.4)	84 (19.4)	150 (33.9)	33 (38.4)
**Socio-economic status**				
Low	811 (19.8)	118 (26.6)	160 (36.6)	38 (44.7)
Medium	2784 (68.1)	285 (64.2)	227 (52.0)	43 (50.6)
High	494 (12.1)	41 (9.2)	50 (11.4)	4 (4.7)
**Lifestyle**				
Physical activity				
Low	839 (19.2)	125 (26.7)	179 (38.6)	34 (40.5)
Light	2719 (62.3)	290 (62.0)	248 (53.5)	43 (51.2)
Moderate	754 (17.3)	52 (11.1)	35 (7.5)	6 (7.1)
High	54 (1.2)	1 (0.2)	2 (0.4)	1 (1.2)
Smoking				
Never	1737 (39.3)	62 (12.9)	146 (30.6)	4 (4.4)
Former	1215 (27.5)	46 (9.6)	102 (21.3)	11 (12.2)
Occasional	179 (4.1)	5 (1.0)	18 (3.8)	2 (2.2)
Daily	1289 (29.2)	368 (76.5)	211 (44.2)	73 (81.1)
Alcohol				
Abstinent	369 (8.6)	61 (13.4)	76 (17.5)	24 (28.9)
Within recommendations	3247 (75.8)	318 (69.6)	284 (64.8)	46 (55.4)
Overuse	667 (15.6)	78 (17.1)	78 (17.8)	13 (15.7)
BMI (kg/m^2)	25.5 (23.1 – 28.3)	25.1 (22.6 – 28.8)	27.2 (23.8 – 30.2)	25.3 (22.1 – 30.0)

Data are presented as median (interquartile range) or n (%).

SRH: self-rated health; suPAR: soluble urokinase plasminogen activator receptor

Supplementary Table 2. Participant's suPAR levels according to self-rated health, and self-rated health according to suPAR levels.

	All	Self-rated health			suPAR		
	n=5490	Excellent/Very good (n=1830)	Good (n=3087)	Fair/Bad (n=573)	Low (0.65 – 2.95 ng/mL, n=1830)	Intermediate (2.95 – 5.46 ng/mL, n=3087)	High (5.46 – 22 ng/mL, n=573)
**Self-rated health**							
Excellent/Very good	1830 (33.3)	1830 (100)	—	—	708 (38.7)	974 (31.6)	148 (25.8)
Good	3087 (56.2)	—	3087 (100)	—	998 (54.5)	1754 (56.8)	335 (58.5)
Fair/Bad	573 (10.4)	—	—	573 (100)	124 (6.8)	359 (11.6)	90 (15.7)
**suPAR (ng/mL)**	3.4 (2.7 – 4.3)	3.2 (2.6 – 4.0)	3.4 (2.8 – 4-3)	3.8 (3.1 – 4.9)	2.5 (2.3 – 2.7)	3.7 (3.3 – 4.3)	6.4 (5.9 – 7.4)

Data are presented as median (interquartile range) or n (%).

suPAR: soluble urokinase plasminogen activator receptor

Supplementary Table 3. Adjusted analyses for the association of self-rated health and suPAR with 2-year hospitalization and 5-year and 15-year mortality according to nine combinations of self-rated health (SRH) and soluble urokinase plasminogen activator receptor (suPAR) levels.

suPAR	SRH	n	2-year hospitalization			5-year mortality			15-year mortality		
			N (%) events	Sub-distribution HR (95% CI)	p	N (%) events	HR (95% CI)	p	N (%) events	HR (95% CI)	p
**Ordered by SRH**											
Low	Excellent/Very good	708	27 (3.8)	ref		2 (0.3)	ref		20 (2.8)	ref	
Intermediate	Excellent/Very good	974	54 (5.5)	1.47 (0.9-2.3)	0.106	10 (1.0)	3.10 (0.7-14.3)	0.148	43 (4.4)	1.34 (0.8-2.3)	0.277
High	Excellent/Very good	148	7 (4.7)	1.24 (0.5-2.8)	0.615	0 (0)	0 (0-0)	<.0001	9 (6.1)	1.52 (0.7-3.4)	0.300
Low	Good	998	60 (6.0)	1.59 (1.0-2.5)	0.046	4 (0.4)	1.28 (0.2-6.9)	0.775	31 (3.1)	0.97 (0.1-1.7)	0.914
Intermediate	Good	1754	166 (9.5)	2.55 (1.7-3.8)	<.0001	26 (1.5)	3.88 (0.9-16.9)	0.072	111 (6.3)	1.71 (1.1-2.8)	0.031
High	Good	335	36 (10.7)	2.89 (1.8-4.8)	<.0001	7 (2.1)	3.97 (0.8-20.5)	0.100	53 (15.8)	3.46 (2.0-6.0)	<.0001
Low	Fair/Bad	124	17 (13.5)	3.77 (2.1-6.9)	<.0001	5 (4.0)	11.32 (2.2-59.4)	0.004	14 (11.3)	3.23 (1.6-6.5)	0.001
Intermediate	Fair/Bad	359	52 (14.5)	4.02 (2.5-6.4)	<.0001	1(0.3)	0.56 (0.0-6.9)	0.648	29 (8.1)	1.81 (1.0-3.3)	0.050
High	Fair/Bad	90	18 (20.0)	5.57 (3.1-10.0)	<.0001	5 (5.6)	9.09 (1.6-61.7)	0.013	16 (17.8)	3.54 (1.7-7.3)	0.001
**Ordered by suPAR**											
Low	Excellent/Very good	708	27 (3.8)	ref		2 (0.3)	ref		20 (2.8)	ref	
Low	Good	998	60 (6.0)	1.59 (1.0-2.5)	0.046	4 (0.4)	1.28 (0.2-6.9)	0.775	31 (3.1)	0.97 (0.1-1.7)	0.914
Low	Fair/Bad	124	17 (13.5)	3.77 (2.1-6.9)	<.0001	5 (4.0)	11.32 (2.2-59.4)	0.004	14 (11.3)	3.23 (1.6-6.5)	0.001
Intermediate	Excellent/Very good	974	54 (5.5)	1.47 (0.9-2.3)	0.106	10 (1.0)	3.10 (0.7-14.3)	0.148	43 (4.4)	1.34 (0.8-2.3)	0.277
Intermediate	Good	1754	166 (9.5)	2.55 (1.7-3.8)	<.0001	26 (1.5)	3.88 (0.9-16.9)	0.072	111 (6.3)	1.71 (1.1-2.8)	0.031
Intermediate	Fair/Bad	359	52 (14.5)	4.02 (2.5-6.4)	<.0001	1(0.3)	0.56 (0.0-6.9)	0.648	29 (8.1)	1.81 (1.0-3.3)	0.050
High	Excellent/Very good	148	7 (4.7)	1.24 (0.5-2.8)	0.615	0 (0)	0 (0-0)	<.0001	9 (6.1)	1.52 (0.7-3.4)	0.300
High	Good	335	36 (10.7)	2.89 (1.8-4.8)	<.0001	7 (2.1)	3.97 (0.8-20.5)	0.100	53 (15.8)	3.46 (2.0-6.0)	<.0001
High	Fair/Bad	90	18 (20.0)	5.57 (3.1-10.0)	<.0001	5 (5.6)	9.09 (1.6-61.7)	0.013	16 (17.8)	3.54 (1.7-7.3)	0.001

CI: confidence intervals; HR: hazard ratios; SRH: self-rated health; suPAR: soluble urokinase plasminogen activator receptor.

Analyses are adjusted for age, sex, smoking, BMI, and comorbidities.
